# Crash and rebound of indigenous populations in lowland South America

**DOI:** 10.1038/srep04541

**Published:** 2014-04-01

**Authors:** Marcus J. Hamilton, Robert S. Walker, Dylan C. Kesler

**Affiliations:** 1 Santa Fe Institute, 1399 Hyde Park Road, Santa Fe, NM, USA and Departments of Biology and Anthropology, University of New Mexico, Albuquerque, NM, USA; 2 Department of Anthropology, University of Missouri, Columbia, MO, USA; 3 Department of Fisheries and Wildlife Sciences, University of Missouri, Columbia, MO, USA

## Abstract

Lowland South America has long been a battle-ground between European colonization and indigenous survival. Initial waves of European colonization brought disease epidemics, slavery, and violence that had catastrophic impacts on indigenous cultures. In this paper we focus on the demography of 238 surviving populations in Brazil. We use longitudinal censuses from all known indigenous Brazilian societies to quantify three demographic metrics: 1) effects of European contact on indigenous populations; 2) empirical estimates of minimum viable population sizes; and 3) estimates of post-contact population growth rates. We use this information to conduct population viability analysis (PVA). Our results show that all surviving populations suffered extensive mortality during, and shortly after, contact. However, most surviving populations exhibit positive growth rates within the first decade post-contact. Our findings paint a positive demographic outlook for these indigenous populations, though long-term survival remains subject to powerful externalities, including politics, economics, and the pervasive illegal exploitation of indigenous lands.

Despite the catastrophic outcomes of European colonization^1^,^2^,^3^,^4^,^5^,^6^,^7^, lowland South America retains a high diversity of indigenous populations. For example, the initial colonization of Brazil by Europeans in the 16^th^ century, resulted in the extinction of ~75% of known societies, the loss of over 95% of the overall population, and the complete collapse of complex urban agricultural societies along coasts and major rivers. Traditionally, lowland South American indigenous cultures were swidden horticulturalists and foragers, but now vary along a spectrum from those almost completely integrated into 21^st^ century market economies to populations with minimal, if any, contact with Western society.

In Brazil, there are a total of 238 contacted indigenous societies, which are censused periodically and results are published by a Brazilian non-governmental organization^8^. These census records cover the last 40–50 years in detail, with some historic population estimates extending back over a century. All of the resulting time-series show evidence of population crashes during the 20^th^ century. Indeed, about one third of the time-series capture contact population crashes of up to 99% mortality, and the remainder demonstrate post-contact exponential growth from small population sizes, almost certainly rarefied by demographic crash events. In addition, the Brazilian government publicizes information for 23 confirmed and 47 potential locations of uncontacted (or isolated) indigenous populations within Brazil, which have been identified most often by brief encounters or aerial surveys. As all uncontacted populations are likely small^2^, a key concern about the future viability of indigenous peoples in lowland South America is the lack of demographic knowledge to best direct conservation policies that promote population viability of recently contacted and isolated peoples. Central to understanding the viability of these populations is to quantify basic demographic parameters and the variables that affect those parameters.

Demographers have long called for better integration of anthropological insights into studies of population dynamics^9^,^10^,^11^,^12^,^13^,^14^,^15^,^16^, including both more intensive fieldwork that accompanies anthropological demography and further incorporation of cultural, political, and historical contexts to yield a more holistic understanding of human demographic outcomes^17^,^18^,^19^,^20^. The extensive nature of the dataset analyzed here provides a unique opportunity to examine the effects of a “natural experiment”, which, in this case is the repeated and well-documented perturbation of a large sample of natural-fertility populations.

Here, we use this rare opportunity to study the long-term population dynamics of a large sample of empirically censused natural-fertility populations and to assess their demographic status. We use the data to measure three key demographic parameters: 1) effects of European contact on indigenous populations; 2) empirical estimates of minimum viable population sizes; and 3) estimates of post-contact population growth rates. We then use this information to conduct population viability analyses^21^ (PVA) of groups undergoing contact. In specific, we use PVAs to assess the probabilities of extinction of these groups over the next 100 years, given the demographic profile of recently contacted groups.

## Results

### (a) Contact effects

**Figure 1:**
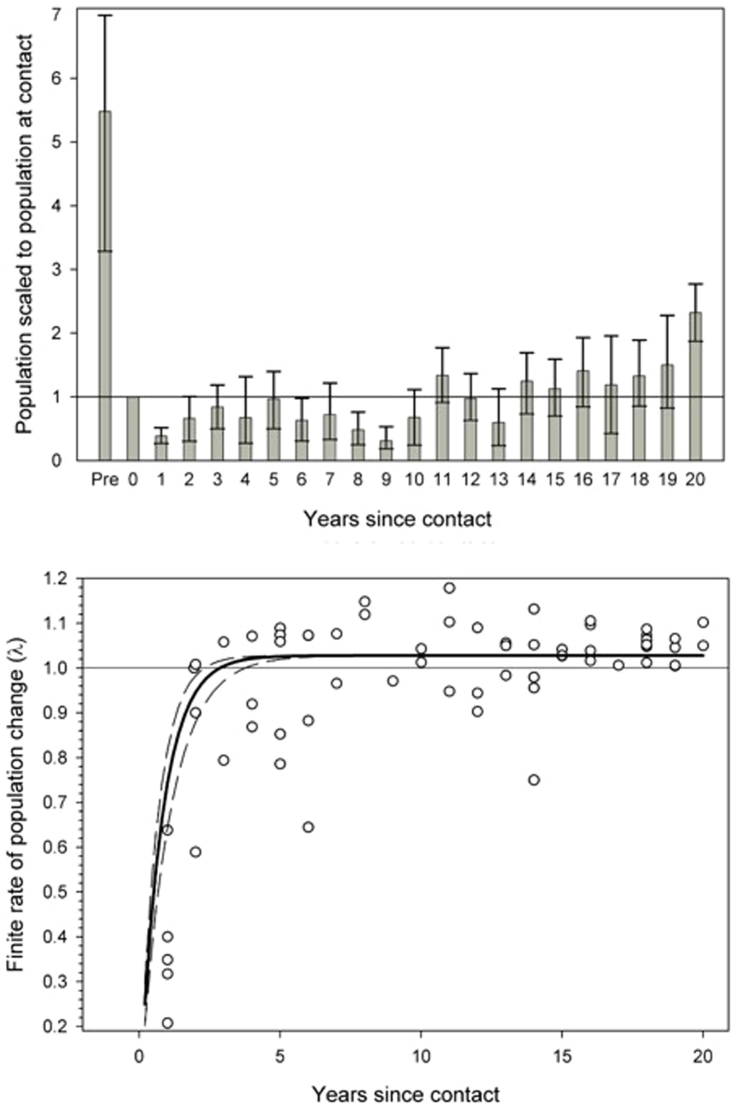
(a) (top). Population sizes scaled to size at initial peaceful contact (year 0, reference line). “Pre” refers to estimates of scaled population sizes before peaceful contact. Bars represent bootstrapped 95% confidence intervals. (b) (bottom). Finite rate of population change as a function of years since contact. Best fit line is a negative exponential with 95% confidence interval of the exponent. Both figures show the devastating effects of contact but suggest that population growth is generally reached within the first decade post-contact.

Our analyses illustrate the devastating effects of contact (Figure 1). Estimates of population sizes before sustained peaceful contact (*n* = 22, recorded an average of 45 years before contact, range 1–106) were on average 5.5 times larger than populations at contact (Figure 1a). Of those time-series that capture contact-related population declines, 3 of 4 declined by more than 80%. The median population size at contact was 245 individuals (*n* = 58). A total of 24 populations with censuses at contact and at least once during the subsequent 20 years had a median contact population size of 189 individuals, and they declined by a mean of 43% from the time of contact. The lowest population numbers were recorded an average of 8.6 years after contact. Whereas some populations begin to recover in the first few years after contact, others exhibited considerable declines during the first decade post-contact (Figure 1b). Fortunately, catastrophic contact effects are relatively short term with most populations rebounding and reaching positive growth rates by the end of the first decade post-contact.

Modeling results of population processes further illustrated the effects of contact. The best approximating model shape was the negative exponential model, which represented a pattern of extreme drop in population immediately after contact, followed by a leveling period, and then finally a growing population. The top-ranked model garnered >99% of the weight, and it out-performed the second-ranked logarithmic model by 46 AIC*_c_* units. The model is represented by the following equation: 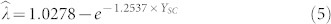
where *Y_sc_* is the number of years since contact (the midpoint of the observation dyad of each calculated 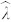). The model represents a precipitous drop in population immediately after contact and during the first several years post-contact. The model suggests that populations stop crashing and begin to grow at 3 years post-contact. Further, the model indicates that after the negative effects of contact have passed, that *λ* asymptotes at ~2.8% annual growth (Figure 1b).

During the analysis phase we tested to see whether absolute year of contact (a proxy of the technological evolution of medicines), and other proxies of access to medicine including distance to major roads and distance to closest town had substantial effects on post-crash population growth rates. None of the effects were significant and so are not reported here. These results indicate that there is no particular time signature to population rebounds, nor a discernable effect of proximity “to the outside world”. However, these results do not necessarily mean that access to healthcare, and the quality of that healthcare play no role in the probability a population rebounds, but that this effect may be challenging to capture empirically in data and measure statistically.

### (b) Minimum population size

**Figure 2:**
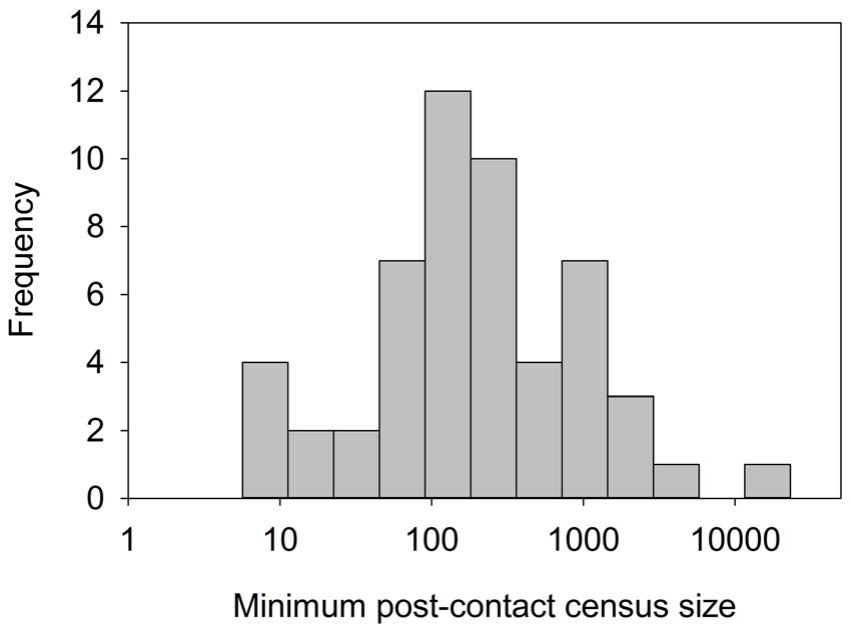
Frequency distribution of all minimum census sizes for all groups observed since or near contact (within 10 years). The distribution is lognormal with a median of 108.

Our data represent complete censuses of populations that survived contact, and thus provide insights into the minimum post-contact population sizes of indigenous populations in Amazonia. In effect, we are asking from what average minimum census size did the populations rebound? The distribution of minimum census sizes of populations observed since contact (or within 10 years) is lognormal, and we operationalize the minimum post-contact population size as the median of this distribution, which is 108 individuals (*n* = 128, Figure 2). This distribution has a large range (2–3,086 individuals), and while several groups (Arikapú, Aruá, and Karipuna of Rondônia) apparently recovered from single-digit numbers, others appear to be teetering on the verge of extinction, including 5 Akuntsu, 4 Juma, and a sole surviving man “Tanaru” with limited contact^2^,^3^. Using the most recent census data available for each group, 20 extant populations are below the median minimum population size, and several of these populations currently exhibit negative population growth rates and are therefore critically endangered. However, given that several populations have recovered from small census sizes, there is at least some recovery potential for currently small populations (including those that remain uncontacted).

### (c) Post-contact population growth rates

**Figure 3:**
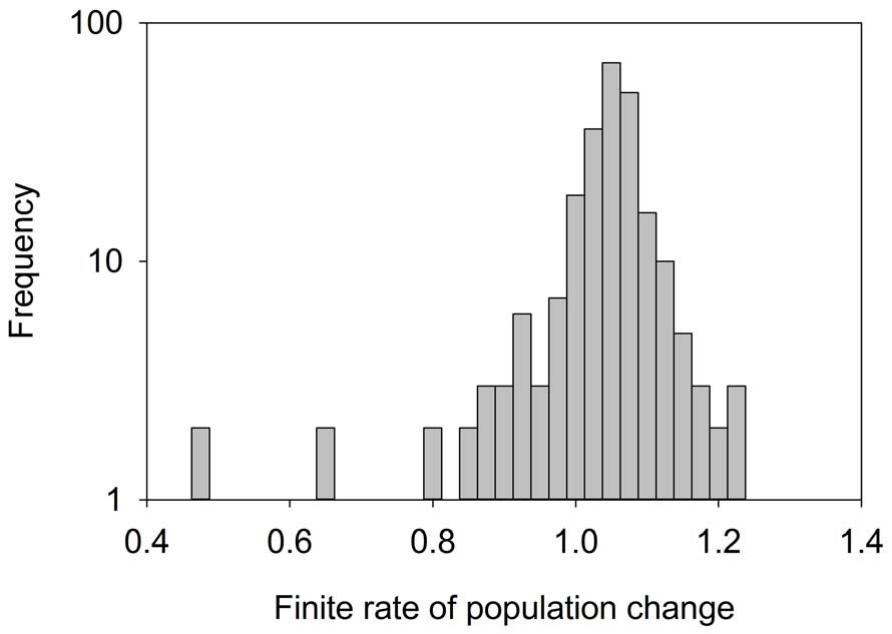
Frequency distribution of the finite rate of population change (λ) from censuses less than 6 years apart and greater than ten years post-contact (median = 4% annual growth). Large declines are mostly disease epidemics, whereas large increases are mostly immigration and group fusioning with previously uncontacted or uncensused populations.

Our results indicate that surviving indigenous populations in the Amazon Basin are remarkably robust and resilient to extrinsic perturbations, with approximately 85% of surviving populations exhibiting net growth over their post-contact time series, most growing at rates among the fastest recorded in any human population (~3–4% annual growth, Figure 3). However, not all of the recorded population growth is reproductive because some increases likely resulted from immigration and group fusioning. High rates of population growth will also occur after disease epidemics preferentially impact old and young individuals (i.e., non-reproductive), as the survivors will have the ability to reproduce quickly.

### (d) Population viability analysis

**Figure 4:**
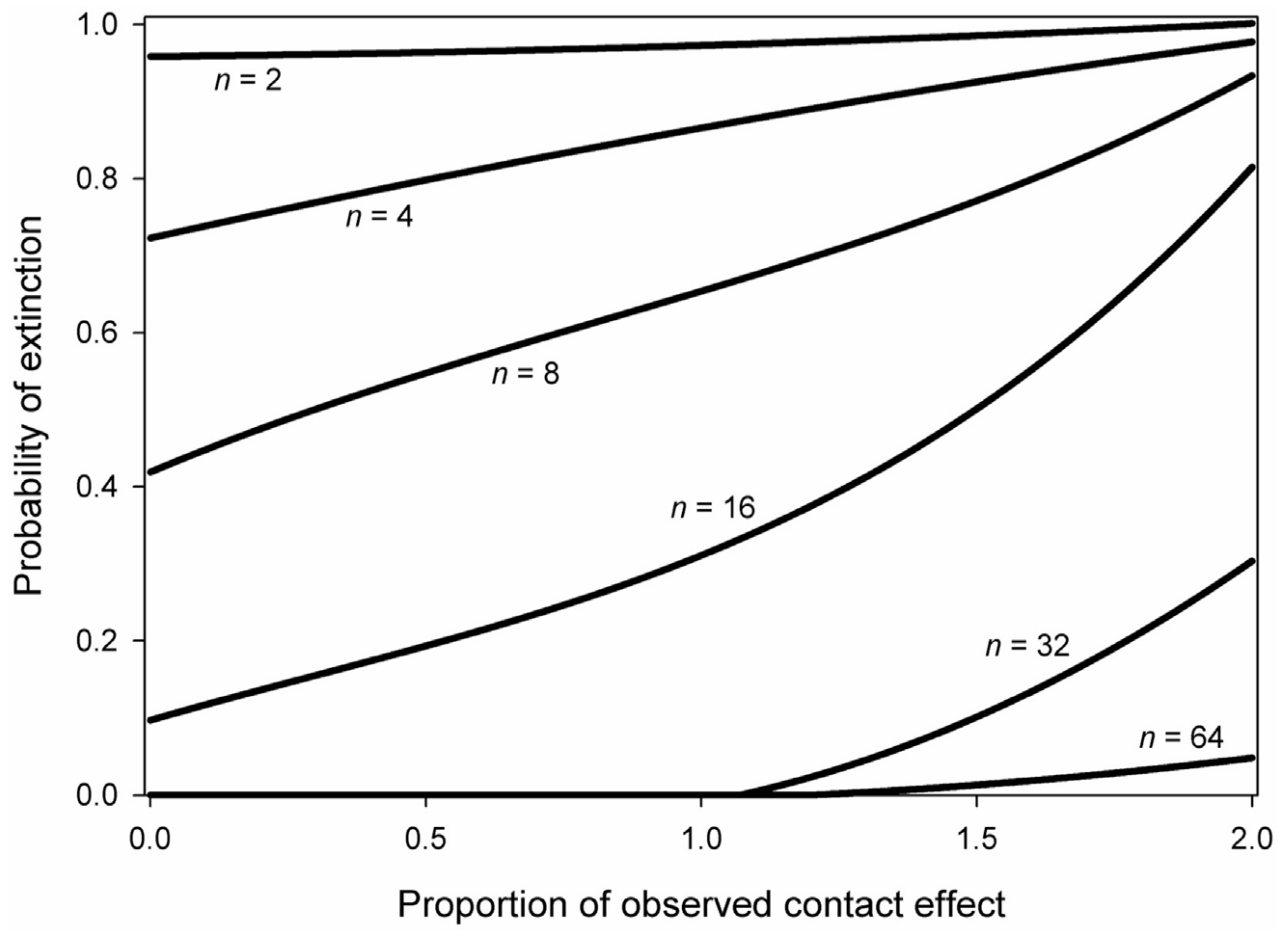
Population viability analysis relating the probability of extinction in 100 years to the proportion of observed contact effect (multiplier of age-specific mortality) from simulation results with loess smoothing. Each line represents contact populations of different sizes (n).

We conducted population viabilty analyses to assess the effects of contact population size and elevated and reduced mortality at contact on the probability of extinction in the 100 years post-contact. Contact population size was the strongest predictor of population persistence, with mean probability of extinction ranging from 98% at a population size of 2, to ~0% with population sizes > 500. The effects of contact (Figure 4), mortality, and fertility were also substantial and interact with population size in such a way that leads to peak effects on extinction with populations of ~15 individuals, with weaker effects at both smaller and larger populations. At extremely small starting population sizes, the effects of contact, mortality, and fertility diminish in importance and demographic stochasticity becomes the primary driver of extinction. In general, a 10% increase in the proportional effect of contact increases the probability of extinction by 4.3%. Mean elasticity values indicate that for each 10% increase in age-specific reproduction, the probability of extinction declined by 7%. For each 10% increase in age-specific mortality, the probability of extinction increased by 5%. Further, the effects of contact differed depending on the age distribution of individuals at the time of contact.

## Discussion

Our analysis dramatically quantifies the devastating effects of European colonization on indigenous Amazonians. Not only did ~75% of indigenous societies in the Brazilian Amazon become extinct, but of the survivors, all show evidence of catastrophic population declines, the vast majority with mortality rates over 80%. However, somewhat surprisingly, our results show that within a decade of peaceful contact most surviving populations rebound extremely fast, exhibiting annual population growth rates of ~4%. Moreover, these growth rates are independent of year of contact and proximity to major roads and towns. Our analyses also suggests that minimum viable population sizes in the Brazilian Amazon are on the order of 100 individuals. Therefore, despite the catastrophic mortality of indigenous Amazonians over the 500+ year contact period, the surviving populations are remarkably resilient and remain demographically viable.

Whereas our data suggest most remaining indigenous Brazilian populations are growing rapidly, the overall long-term health and conservation of these societies is far from certain due to increasing habitat loss; the breakdown of essential indigenous metapopulation structures; increasing acculturation with the outside world; the loss of cultural knowledge held by older generations; and persistent health disparities^1^,^2^,^3^,^4^,^5^,^6^,^7^,^27^,^28^. Although severe crashes associated with contact appear to be ubiquitous, rebound growth is fast, and many populations continue to increase exponentially as they are likely well below carrying capacity. Population viability analyses suggest that better health care and other policies that alleviate the negative effects of contact by elevating survival and increasing fertility at the time of contact are most efficient for populations of around 10 to 20 individuals, which may characterize some of the perhaps 100 or so uncontacted societies remaining in lowland South America^2^. Our results have positive implications for the long-term survival of currently isolated (i.e., uncontacted) populations, even if they are comprised of only small numbers of individuals, provided they can survive population losses occurring before and after contact and they are allowed to maintain access to protected habitats large enough to support subsistence needs and future population growth.

While our study was limited to the contact period of the Brazilian Amazon, our results suggest that natural-fertility human populations in the past likely had the ability to rebound quickly from population bottleneck events that rarefied populations to a fraction of their former size. This rebound ability is even more likely in pre-contact eras where indigenous habitats would not have been under the additional suite of external pressures we see in lowland South America today, such as both legal and illegal logging, mining, and squatting.

## Methods

Population data were obtained from Ricardo and Ricardo^9^,^10^,^11^,^12^,^13^,^14^ and from the accompanying website of the Instituto Socioambiental (http://pib.socioambiental.org). Each record included ethnolinguistic names, estimated population sizes, geographic coordinates for each group, language family, the year of contact, and the year that estimates were made. We classified populations as uncontacted until sustained peaceful contact had been made with neo-Brazilians, missionaries, or government workers. We excluded groups with populations straddling international borders because census counts did not include portions in other countries (*n* = 43).

We used sequential censuses of population observations to estimate the finite rate of population change, *λ*^27^, for each group for which multiple population estimates were available during the first 20 years after contact, using the following formula: 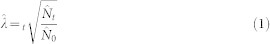
where *N_0_* is the population size at the beginning of the interval, and *N_t_* is the population size at the conclusion of the interval, and t is the number of years encompassed. We excluded groups with <2 observations and census intervals >6 years apart to avoid biased estimates. This resulted in a dataset comprised of 24 different ethnolinguistic groups with 67 total estimates of λ between the years 1923 and 2010.

We evaluated the effects of contact on λ during the first twenty years post-contact (approximately the first generation) with generalized nonlinear mixed models. We first considered 5 primary model forms: null (*λ = β_0_*), linear relationship (*λ = β_0_ + β_1_Y_sc_*), first order quadratic relationship (*λ = β_0_ + β_1_Y_sc_* *+ β_2_Y_sc2_*), inverse logarithmic relationship (*λ = β_0_ + β_1_*ln(*Y_sc_*)), and a negative exponential relationship (*λ = β_0_ − e^β1Ysc^*). Models included a response variable (*λ*), the associated fixed effect variable of years since contact (*Y_sc_*) for the interval midpoint, and a random intercept variable identifying each group. Data were fitted with nonlinear mixed models (proc nlmixed in SAS 9.2, Carry, NC), and results were used to rank models with an information theoretic approach, such that the model with the lowest adjusted Akaike information criterion (AIC_c_) and greatest model weight was considered best approximating^22^ and used in population modeling.

The PVA was conducted using program Vortex (http://vortex9.org), and the parameterization was based on results of the first analysis phase, and on information from the literature. Simulations were based on 100 year scenarios, and no genetic or inbreeding effects were included. Age-specific survival was estimated using the Siler model^23^: 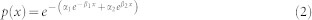
where *x* represents age in years, *α_1_* was set to 0.309, *β_1_* was set to 1.138, *α_2_* was set to 0.009, and *β_2_* was set to 0.09. These parameter estimates are from averages from small-scale human societies^24^. The reproduction function was derived from the total number of live births for females, which is 5.8 across 81 small-scale societies^25^ (http://dice.missouri.edu). The age-specific proportion of women birthing between the ages of 12 and 45 was taken from the well-studied forest Ache^26^ and adjusted to give a total fertility rate of the mean of 5.8 and fit with a third-order quadratic equation to yield age-specific births: 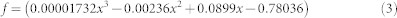
where *f* is the number of offspring expected for females of age *x* (*r^2^* = 0.98). Males became reproductive at 15. All females were restricted to one birth each year.

The effects of contact were added to the PVA model by adjusting survival for the first twenty years of simulations. Survival was adjusted across all ages with a multiplicative adjustment parameter, so that the effects of contact were proportional across age classes. The shape of the underlying Siler survival model yielded a net result with larger numbers of young and old individuals impacted by contact than those at middle ages because the already depressed survival in those age groups was exaggerated. Survival adjustment parameters were estimated by structuring an age-based population matrix that incorporated the above vital rates. The female-based matrix was composed with an annual time step and with single-year age elements. We used MATRIX 2.3 (Foxes Team, Rome, Italy) to identify the dominant eigenvalue for the matrix, which represents the asymptotic finite rate of population change (*λ*). The survival adjustment parameter was then altered manually until the matrix yielded *λ* values that aligned with the value predicted by the top-ranked model from the analysis of contact effects. Adjustment parameters were estimated for the first 20 years post-contact and incorporated into the Vortex simulation model to represent the effects of contact on population change.

The fully parameterized Vortex model was then used in a simulation analysis to evaluate the potential effects on population persistence of differential contact population sizes, of mortality, of fecundity, and of increased or decreased contact effects. Population persistence was measured for the first 100 year after contact, and populations were considered to go extinct if they were reduced to a single individual. Contact population sizes from 2–1024 individuals in doubling increments were considered for all contact, mortality, and fecundity scenarios with an additional 10 even-numbered simulations for contact population sizes between 10 and 28. The effects of perturbed contact effects and vital rates were evaluated with multipliers that elevated and depressed each variable. For example, the Siler equation predicted mortality of approximately 4% at age 2, which was elevated in simulations with a multiplier of 1.5 to 6%. In the same simulation, predicted mortality of a 20-year old was elevated with the same multiplier from approximately 1% to 1.5%. A similar age-specific multiplier was applied to reproduction in the simulated populations. Each combination of input variables was used for 1,000 simulations, whereon we recorded the proportion of simulated populations that went extinct, the median time to extinction, and the mean population size of surviving populations at the conclusion of the 100-year simulation time frame. To provide insights into the changes in the probability of population persistence that are brought about by proportional changes in each of the population process metrics we presented elasticity metrics^22^: 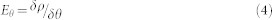
where *θ* is the parameter of interest, *δ* is the proportional change in each parameter and *ρ* is the probability of extinction.

## Author Contributions

M.J.H., R.S.W. and D.C.K. conceived of, and wrote the paper. Figures 2a and b, and 5 generated by D.C.K., and figures 3–4 generated by M.J.H. The data set was compiled by M.J.H. and R.S.W., and population viability analysis was conducted by D.C.K. All authors reviewed the manuscript.
